# Cell–Cell and Cell–Matrix Interactions at the Presumptive Stem Cell Niche of the Chick Corneal Limbus

**DOI:** 10.3390/cells12192334

**Published:** 2023-09-22

**Authors:** Kiranjit K. Bains, Robert D. Young, Elena Koudouna, Philip N. Lewis, Andrew J. Quantock

**Affiliations:** Structural Biophysics Group, School of Optometry and Vision Sciences, Cardiff University, Maindy Road, Cardiff CF10 3AT, UK; bainsk@cardiff.ac.uk (K.K.B.); youngrd@cardiff.ac.uk (R.D.Y.); koudounae@cardiff.ac.uk (E.K.); lewispn@cardiff.ac.uk (P.N.L.)

**Keywords:** cornea, stem cell niche, scanning electron microscopy, transmission electron microscopy, volume electron microscopy, serial block-face scanning electron microscopy

## Abstract

(1) Background: Owing to its ready availability and ease of acquisition, developing chick corneal tissue has long been used for research purposes. Here, we seek to ascertain the three-dimensional microanatomy and spatiotemporal interrelationships of the cells (epithelial and stromal), extracellular matrix, and vasculature at the corneo-scleral limbus as the site of the corneal stem cell niche of the chicken eye. (2) Methods: The limbus of developing (i.e., embryonic days (E) 16 and 18, just prior to hatch) and mature chicken eyes was imaged using scanning electron microscopy (SEM), transmission electron microscopy (TEM), and the volume electron microscopy technique, serial-block face SEM (SBF-SEM), the latter technique allowing us to generate three-dimensional reconstructions from data sets of up to 1000 serial images; (3) Results: Data revealed that miniature limbal undulations of the embryonic basement membrane, akin to Palisades of Vogt (PoV), matured into distinct invaginations of epithelial cells that extended proximally into a vascularized limbal stroma. Basal limbal epithelial cells, moreover, occasionally exhibited a high nuclear:cytoplasmic ratio, which is a characteristic feature of stem cells. SBF-SEM identified direct cell–cell associations between corneal epithelial and stromal cells at the base of structures akin to limbal crypts (LCs), with cord-like projections of extracellular matrix extending from the basal epithelial lamina into the subjacent stroma, where they made direct contact with stomal cells in the immature limbus. (4) Conclusion: Similarities with human tissue suggest that the corneal limbus of the mature chicken eye is likely the site of a corneal stem cell niche. The ability to study embryonic corneas pre-hatch, where we see characteristic niche-like features emerge, thus provides an opportunity to chart the development of the limbal stem cell niche of the cornea.

## 1. Introduction

The corneal epithelium uses a combination of homeostatic and reparative processes to maintain its functional integrity, a vital requisite for good visual acuity. As with most self-renewing tissues, stem cells are regulated by a microenvironment commonly referred to as a niche. For the cornea, the widely accepted hypothesis is that epithelial and mesenchymal stem cell populations are located at the limbus, the transition zone between the cornea and sclera. The limbal niche is believed to comprise a unique matrix composition and microstructure that provides a range of cues and sustenance to the resident stem cell populations to meet the regenerative demands of the ocular surface at any given time [1]. The corneal limbus has become an area of interest to elucidate the biological mechanisms and cell–cell interactions that regulate cell stem cell behavior and can be used to characterize shared anatomical features across species.

In the human cornea, radially oriented “interpalisadal epithelial ret ridges” of stroma corresponding to limbal palisades of Vogt (PoV) were first described by Goldberg and Bron [2] and seen to predominate at the upper and lower aspects of the limbal arc. Epithelial-cell-filled crypts between PoV were later termed limbal crypts (LCs) by Shortt and associates [3], who described them as “distinct invaginations of epithelial cells extending from the peripheral corneal epithelium into the corneal limbal stroma”. It has also been reported that PoVs are highly cellularised and inclusive of a distinct vascular supply, and that some basal cells contained within LCs are unusually small with a higher nuclear:cytoplasmic ratio than the majority of epithelial cells that line the bases and edges of LCs.

Although there has been a concerted effort to characterize the limbal stem cell niche in humans, only a limited number of studies have attempted to identify an apparent limbal niche in other animals and categorize any species-related differences that may exist [4,5,6,7]. Here we identified, as in the human limbal stem cell niche, anatomical features akin to PoV and LCs [2,3,8,9,10,11] in the mature avian limbus, as well as evidence of direct cell–cell interactions between basal epithelial cells and stromal cells [9]. The chicken model has long been used to investigate corneal development [12,13,14,15,16,17] and has proven to be an effective model for understanding corneal repair mechanisms [18,19]. The implications of our findings suggest new avenues for future work using the chicken eye to understand the development of the corneal stem cell niche and its application in developing new disease models to study the mechanisms that affect limbal function, which can lead to disorders like limbal stem cell deficiencies.

## 2. Materials and Methods

### 2.1. Tissue Acquisition

**Figure 1:**
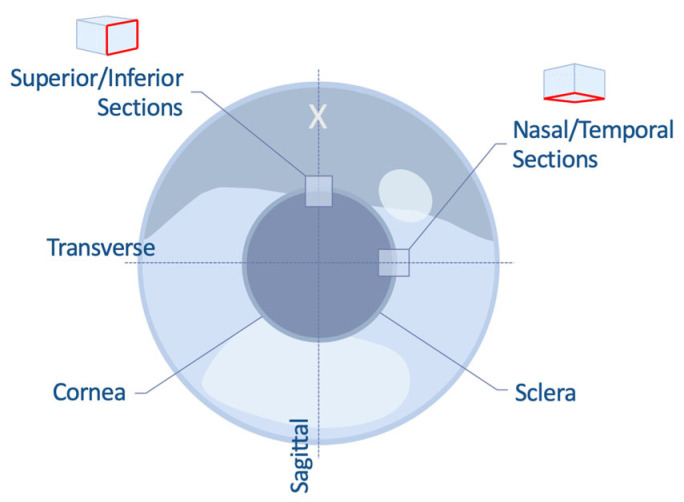
Schematic representing the dissection of embryonic chicken corneas for SBF-SEM and TEM. A reference incision point (X) was made with a scalpel blade while the eye was in situ to aid in the correct orientation of the eye post-removal. Corneal sections 2 mm wide were cut along the sagittal and transverse planes to produce superior/inferior or nasal/temporally oriented sections containing the limbus. Red outlines correspond to the cutting face.

Whole embryonic eyes were harvested from fertilized, white leghorn chicken eggs (*n* = 6; Henry Stewart & Co. Ltd., Fakenham, UK) incubated to days 16 and 18 of embryogenesis (E16 and E18), followed by termination of embryos using cervical decapitation and subsequent dissection of whole eyes from the orbital cavity. Whole adult eyes were extracted from JA87 chicken heads obtained within hours of slaughter from a local abattoir (*n*=12; Capestone Organic Poultry Ltd., Haverfordwest, UK). Corneas with a rim of sclera intact were dissected for scanning electron microscopy (SEM). For serial block-face (SBF) SEM and transmission electron microscopy (TEM), superior/inferior, or nasal/temporally oriented sections (5 mm long by 2 mm wide in the radial direction) were excised from the limbal region (Figure 1). Information as to the orientation of the adult corneas (i.e., superior and inferior vs. nasal and temporal) was not available.

### 2.2. Scanning Electron Microscopy

Corneoscleral discs from adult chickens were fixed in 2% paraformaldehyde (PFA)/2.5% glutaraldehyde in 0.1 M sodium cacodylate buffer (pH 7.4) for 24 h at room temperature. They were then washed in 0.1 M sodium cacodylate buffer and processed using a modification of the method described by Komai and Ushiki [20] and Thale and Tillmann [21]. Tissue maceration was carried out by immersing specimens in 10% sodium hydroxide over a course of 5 days at room temperature. Samples were then washed and stored in distilled water for 24 h before being transferred into a solution of 2% tannic acid for 5 h, washed once more in distilled water, and then immersed for 2 h in aqueous 1% osmium tetroxide. After another wash with distilled water, tissue segments underwent dehydration using a series of ethanol treatments from 70% to 90% (both 1 × 30 min) and 100% (2 × 30 min). The tissue was then dried using hexamethyldisilazane (HMDS, Alfa Aesar, UK) by transferring it into a 1:1 solution of 100% ethanol and HMDS for 1 h, followed by 2 × 1 h treatments in 100% HMDS. The tissue was then transferred to fresh HMDS and left uncovered in a fume hood until all the liquid had evaporated. After, it was mounted onto 12.5 mm aluminum stubs using carbon cement and coated with ~15 nm of gold-palladium using a BioRad SC500 sputter coater with argon as the sputtering gas. The tissue was then imaged using a Zeiss Sigma HD field emission gun scanning electron microscope (Carl Zeiss Ltd., Cambridge, UK). A beam energy between 5 and 10 kV was used, with a 30 µm final aperture and a nominal beam current of ~150 pA. Working distances ranged between 5.1 and 48.5 mm, and images were acquired between 21 and 40,000× magnification.

### 2.3. Serial-Block Face Scanning Electron Microscopy

Dissected segments of limbal tissue were fixed in 2% PFA/2.5% glutaraldehyde in 0.1 M sodium cacodylate buffer (pH 7.4) overnight at 4 °C and processed using a modification of the method described by Deerinck et al. [22] to enhance cell and tissue contrast. Initially, segments were immersed in a solution of 1.5% potassium ferricyanide/1% osmium tetroxide for 1 h, followed by a brief wash in distilled water. The samples were then transferred to 1% aqueous thiocarbohydrazide for 30 min and again washed in distilled water before successive 1 h immersions in 1% osmium tetroxide, followed by 1% aqueous uranyl acetate, and a final wash in distilled water. Specimens were then immersed in Walton’s lead aspartate for 1 h at 60 °C, washed, and serially dehydrated in ethanol by immersion in 70% and 90% solutions (both 1 × 15 min), followed by 100% (2 × 15 min). After 2 × 15 min immersions in propylene oxide, specimens were placed overnight in a 1:1 mixture of propylene oxide and Araldite CY212 epoxy resin without a benzyl dimethylamine (BDMA) accelerator. The next day, after a series of five changes in Araldite resin without BDMA, the tissue underwent a series of seven changes with Araldite resin, including BDMA, over the course of 48 h. Specimens were then oriented in embedding molds containing resin such that the radial surface was exposed for imaging and subsequently polymerized at 60 °C for 24 h. All solutions and distilled water were passed through Minisart^®^ syringe filters (Sartorius Stedim Biotech GmbH, Goettingen, Germany), and, unless specified, all reagents and equipment were supplied by Agar Scientific Ltd. (Stansted, UK). Treatment was at room temperature unless stated.

Resin blocks were cut and glued to aluminum pins before being polished using ultramicrotomy and coated with ~10 nm of gold-palladium using an ACE 200 sputter coater (Leica Microsystems, Milton Keynes, UK). Samples were transferred into the chamber of a Zeiss Sigma VP Field Emission Gun scanning electron microscope (Carl Zeiss Ltd., Cambridge, UK), where the block face was imaged at 3.5 KV at a pixel resolution of 4 nm and using a dwell time of 8 μs. For each specimen, a series of 550–1000 images of the block face were acquired, each alternating with the removal of a 100 nm slice of tissue from the block surface by the in-chamber diamond-knife ultramicrotome. All SBF-SEM data sets were recorded at 4096 × 4096 (4 K) in Gatan format dm4 files, and these were subsequently batch converted to “.tiff” format. The converted data were then exported into Amira software (version 2020.2, ThermoFisher Scientific, UK) to generate 3D reconstructions of each SBF-SEM data set. The limbal ultrastructure was reconstructed and rendered in 3D using a combination of surface generation (isosurface) and semi-automated 3D volume generation (Volren). Manual segmentation was used to segment specific ultrastructural elements in situations where insufficient contrast precluded an automated approach. Videos of 3D reconstructions, saved as “.mpg” movies, were produced using the “Animation” feature to generate a 360° view of the reconstruction.

### 2.4. Transmission Electron Microscopy

Limbal tissue segments were processed into resin blocks as described in the previous section, after which semi-thin sections (300 nm thick) were cut on a UC6 ultramicrotome (Leica Microsystems, Milton Keynes, UK) and stained with toluidine blue for light microscopy to identify appropriate regions of interest. Ultrathin sections, <100 nm thick, were then cut using a diamond knife and collected on uncoated G300 copper electron microscopy grids (Gilder Grids, Grantham, UK). The sections were imaged using a Jeol 1010 transmission electron microscope (Jeol (UK) Ltd., Welwyn Garden City, UK) operating at 80 kV.

## 3. Results

### 3.1. Anatomical Features of the Adult Chicken Limbus

**Figure 2:**
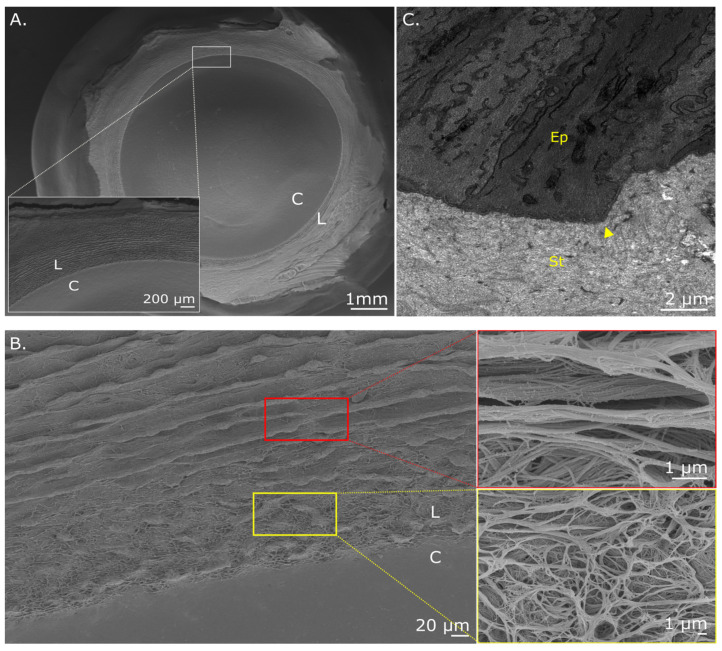
Scanning and transmission electron microscopy of the adult chicken corneoscleral disc. (**A**) En face view of the adult cornea (C) showing a distinct demarcation between the limbal zone (L), which can be seen extending circumferentially around the cornea. (**B**) At higher magnification of the edge of the cornea, the tissue surface has a porous texture (yellow box) with distinct but non-uniform, concentric ridge and furrow projections (red box) running parallel to the limbal boundary. (**C**) TEM of the epithelial basement membrane shows an abrupt change in basement membrane orientation at the limbus (arrowhead). Epithelium (Ep); Stroma (St). The cornea is to the left of the image.

SEM observations of the surface of the adult chicken corneoscleral disc from which the epithelium had been removed by cell maceration revealed a distinct margin at the transitional zone between the peripheral cornea and limbus that extended circumferentially around the cornea (Figure 2A). The peripheral cornea appeared flatter and smoother compared to the scleral side of the limbus, where non-uniform, concentric ridges and furrow-like projections parallel to the marginal line were seen (Figure 2B). At higher magnification, the concentric projections displayed two apparent zones that differed in their anatomical identity. The inner belt (closest to the peripheral cornea) was reminiscent of the human anterior limbal cribriform layer with a highly porous and irregular network of thin fibers interspersed between and around contorted ridge projections (Figure 2B, yellow box). The outer zone, on the other hand, was comprised of a compact network of fibers located at the furrows of the concentric projections (Figure 2B, red box). Both ridge and furrow projections were continuous around the entirety of the cornea and aligned with the identifiable curve of the cornea edge. Similar observations of the differing fiber networks along the limbal zone were evident in SEM images taken of the E16 and E18 corneas. TEM also highlighted the profile of the basement membrane as it transitions from the peripheral cornea into the limbus (Figure 2C). An abrupt change can be seen, switching from a regular uniform contour between the epithelium and underlying stroma in the peripheral cornea to an irregular profile at the limbus, which results in an apparent increase in the area of interaction between epithelial basal cells and the underlying stromal extracellular matrix.

**Figure 3:**
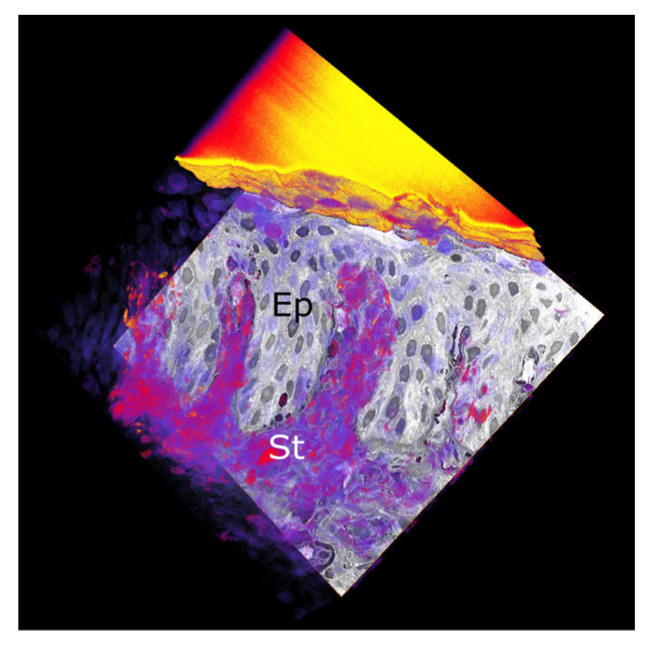
SBF-SEM of the adult chicken corneoscleral limbus. Limbal reconstruction from a SBF-SEM dataset of adult limbus shows undulating folds or finger-like projections of the epithelium (Ep) and superficial stroma (St). The gold-coloured block overlies the surface of the limbal epithelium.

Light microscopy of the adult chicken limbus often revealed distinct undulating folds of upward (stromal) and downward (epithelial) projections. To better understand this microanatomy in 3D, the limbal region was studied by SBF-SEM. Rendered and reconstructed 3D representations from image sequences at 100 nm resolution indicated the presence of numerous finger-like undulations within the corneolimbal junction (Figure 3). The examination of each quadrant location suggests this ultrastructure may be a feature around the whole circumference of the limbus, as evidenced in SBF-SEM videos representative of different limbal quadrants (Supplementary Videos S1 and S2).

**Figure 4:**
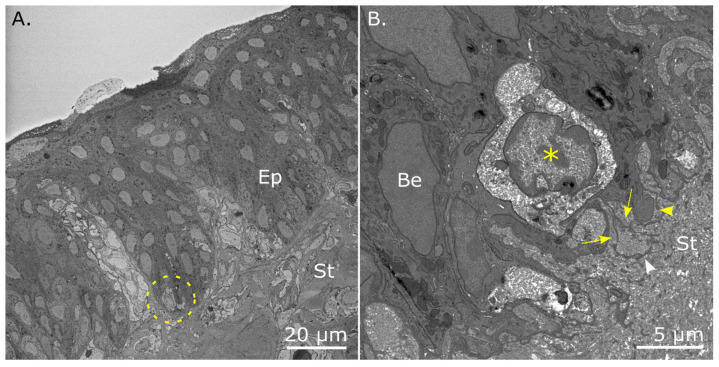
Basal epithelial cells with a high nuclear-to-cytoplasmic ratio within the presumptive limbal crypt of the adult chicken cornea. (**A**) An image of the block face taken from a SBF-SEM dataset shows a limbal epithelial cell (dashed circle), which is morphologically smaller compared to neighboring basal cells with a high nuclear-to-cytoplasmic ratio, a barely detectable nucleolus, and euchromatin as open DNA. (**B**) The TEM of a limbal basal epithelial cell (Be) surrounds a smaller and rounder basal cell (asterisk), exhibiting a high nuclear-to-cytoplasmic ratio. Lobed basal protrusions (yellow arrowhead) with associated basal lamina are also seen to project downward into the underlying superficial stroma (St). Cytoplasmic extensions (arrows) can be seen extending from a mesenchymal cell (white arrowhead) to contact the basal lamina and insert between basal cell protrusions.

Individual sections from SBF-SEM datasets of the limbus revealed that occasional basal epithelial cells within the downward projections of the epithelium appeared morphologically different from neighboring basal epithelial cells. As seen in Figure 4, these cells were comparatively smaller and rounder than those surrounding them and had a higher nuclear:cytoplasmic ratio, which is a characteristic feature of stem cells. Moreover, these cells possessed a barely detectable nucleolus and lightly packed euchromatin. TEM also revealed that basal cells formed bulbous lobed protrusions into the underlying stroma (Figure 4B) which maintained an intact basal lamina across the contour of the limbal zone. Cytoplasmic extensions were also seen to extend from mesenchymal cells to form direct cell-to-cell interactions with the basal lamina and insert between lobed basal cell protrusions.

**Figure 5:**
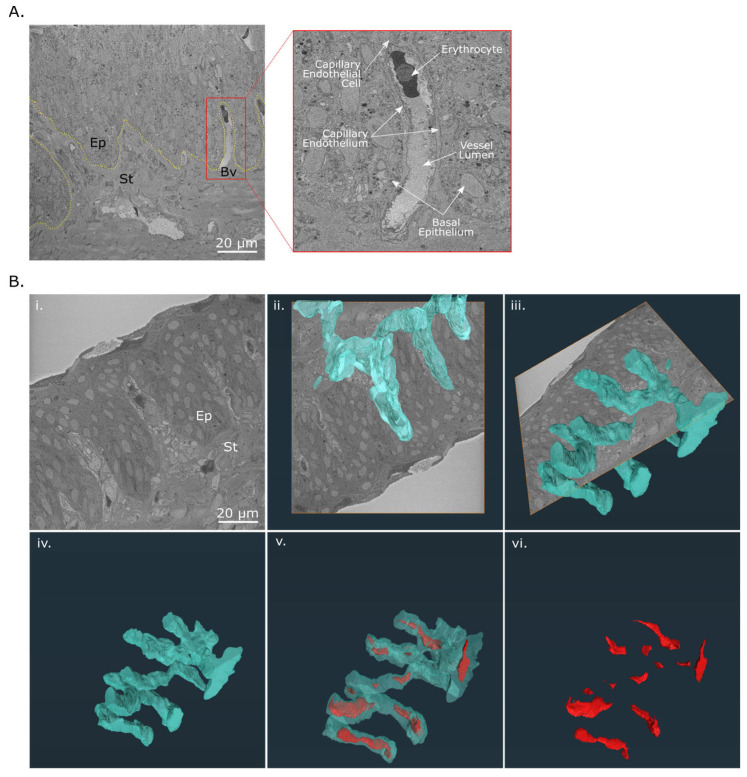
Network of limbal blood vessels in the adult chicken cornea. (**A**) Cross-section of the limbus taken from a SBF-SEM dataset showing a blood vessel (Bv, red box) in the upward projection of the stroma (St) into the epithelium (Ep). The blood vessel is surrounded by endothelial cells and possesses a vessel lumen containing a nucleated blood corpuscle. (**B**) The 3D-reconstructed network of blood vessels located within the upward stromal projections of the adult corneal limbus. (**i**) A raw backscatter electron image of the adult limbal zone with superimposed 3D reconstructed blood vessels (blue volume) oriented along the sectional plane (**ii**) and at a rotated view (**iii**) was generated from a serial data set. (**iv**–**vi**) Red blood corpuscles (red volume) are reconstructed within the lumen of the blood vessels. The dataset is viewable in Supplementary Video S3.

Vessel-like structures, seen within upward stromal projections, were further investigated using SBF-SEM. The 3D volume data demonstrated the presence of a blood vessel surrounded by capillary endothelial cells. Contained within the vessel lumen was a nucleated red blood corpuscle (Figure 5A). A 3D reconstruction of the vascular supply revealed a complex network of interconnected branches, perpendicular to the sectional plane, within the stromal projections that extended throughout the limbal zone. It also contained remnants of red blood corpuscles (Figure 5B).

### 3.2. Anatomical Features in the Embryonic Avian Limbus

**Figure 6:**
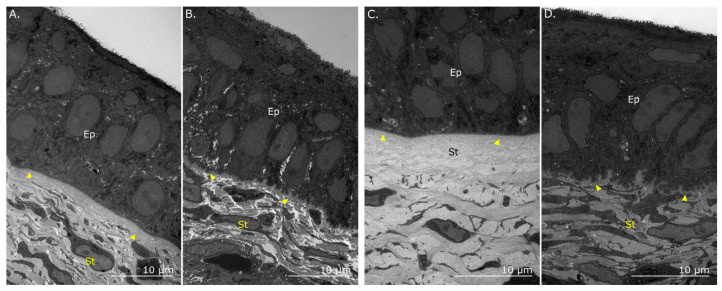
Transmission electron microscopy of the chicken epithelial basement membrane in the embryonic limbus and peripheral cornea. The peripheral cornea at E16 (**A**) and E18 (**C**) contains a distinct and uniform basement membrane (arrowheads) between the corneal epithelium (Ep) and stroma (St), which becomes undulated and non-uniform at the limbus at embryonic days 16 ((**B**), arrowheads) and 18 (（**D**), arrowheads).

Like the adult chicken cornea, the basement membrane of the E16 (Figure 6A) and E18 (Figure 6C) corneas in the corneal periphery remained uniform and regular, with evidence of a subjacent, acellular Bowman’s layer. However, at the limbal junction for both embryonic stages, the basement membrane, now without a visible Bowman’s layer, contained far more frequent undulations (Figure 6B,D). These, however, were comparatively less pronounced than the longer finger-like projections seen in the adult limbus. In the embryonic limbus, no cells with a noticeably high nuclear-to-cytoplasmic ratio were detected by SBF-SEM within basal cells in the limbal epithelium.

### 3.3. Cell–Cell Interactions in the Embryonic Avian Limbus

**Figure 7:**
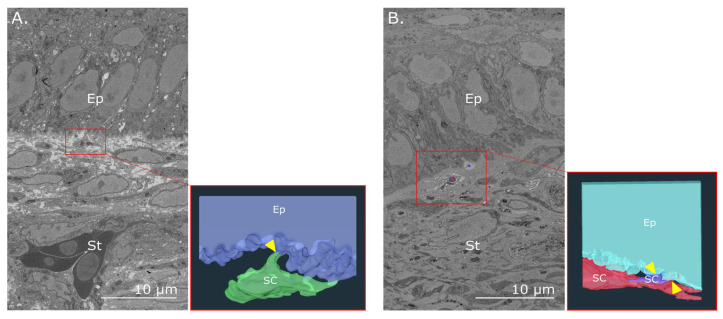
Direct interaction between limbal stromal and basal epithelial cells in the embryonic chick cornea. An SBF-SEM image of the limbus at E16 (**A**) and E18 (**B**) taken from the block face of separate SBF-SEM datasets shows a stromal cell (SC, red box) near a basal epithelial cell. Corresponding 3D reconstructions show an anterior view of a stromal cell at E16 extending a process to directly interact (arrowhead) with a basal epithelial cell (**A**) and two stromal cells at E18 directly interfacing (arrowheads) with a basal epithelial cell (**B**). Ep—epithelium; St—stroma.

**Figure 8:**
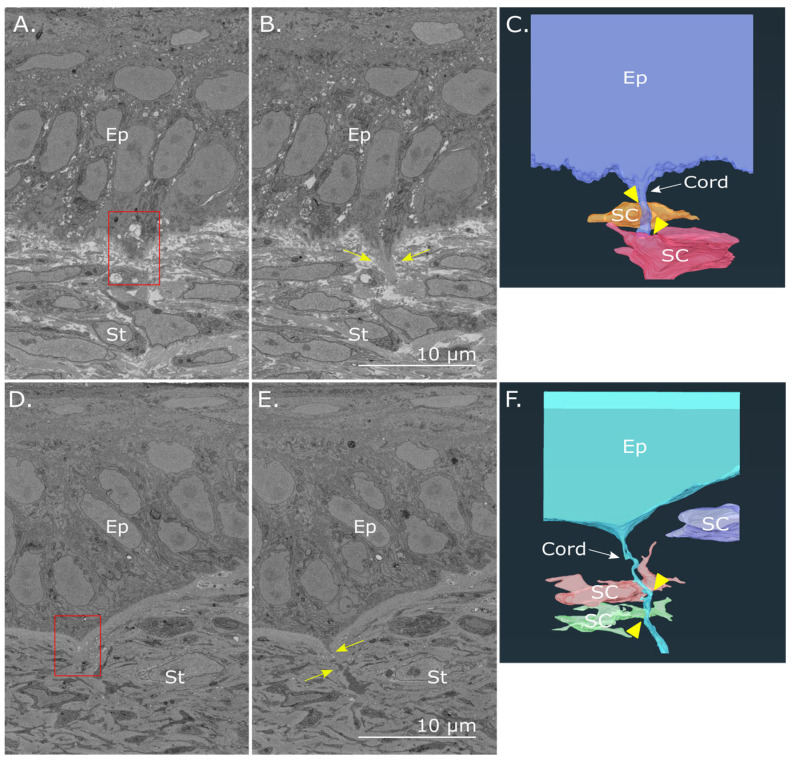
Extracellular matrix “cord” projection in the embryonic chicken cornea. Serial SBF-SEM images of the peripheral cornea at E16 (**A**,**B**) and E18 (**D**,**E**) were taken of the block faces of individual SBF-SEM datasets. A single matrix “cord” projection is seen emerging from a protrusion ((**A**,**D**), red box) in the epithelial basal lamina and extending proximally into the stromal matrix ((**B**,**E**), arrows). 3D reconstructions of a matrix “cord” projecting from the basal epithelium at E16 (**C**) and E18 (**F**) are seen to interface (arrowheads) with stromal cells (SC) in the underlying cornea (St). Ep —epithelium. Datasets are viewable in Supplementary Videos S4 and S5.

Observations of the corneoscleral limbus in E16 (Figure 7A) and E18 (Figure 7B) chick corneas showed anterior stromal cells in close proximity to basal epithelial cells. Consecutive 3D reconstructions demonstrated evidence of cell–cell interactions taking place along the basement membrane via stromal cell processes that extended proximally to form focal contact with associated epithelial cells. Additionally, “cords” of extracellular matrix were seen to emerge from limbal protrusions in the epithelial basal lamina that projected deeper into the stroma at both E16 (Figure 8A,B) and E18 (Figure 8D,E). The 3D reconstructions also showed cords interfacing with multiple stromal cells directly below the basement epithelium in E16 (Figure 8C) and E18 samples (Figure 8F).

## 4. Discussion

### 4.1. Adult Chicken Corneal Limbus

The corneal limbus is widely accepted as being a supportive microenvironment for both epithelial and mesenchymal stem cell populations in the eye. Using SEM, we examined the surface of the adult chicken limbus, following removal of the corneal epithelium by chemical maceration, for microstructures previously identified in the human limbus by Shortt et al. [3], such as PoV, LCs, and focal stromal projections (FSP). Clinically, human PoV and LCs can be viewed using an ophthalmic slit lamp microscope, whereas FSPs are obscured by overlying stratified epithelium. These microstructures were indiscernible along the avian limbus with light microscopy on intact eyes, also providing no evidence of radially extending structures akin to LCs and PoV seen in the human limbus by Dziasko and colleagues [9]. We speculate that interpalisadal ridges may be obscured in avian eyes due to lighter pigmentation arising from lower concentrations of melanin-containing cells compared to human eyes, as observed by Goldberg and Bron [2]. Alternatively, tissue maceration with sodium hydroxide during sample processing for SEM may fail to preserve the avian interpalisadal ridges, although these structures remained visible in the human eye despite decellularization of the corneal surface when examined by Short and colleagues [3]. SEM observations in the present study thus suggest an absence of these structures in the avian limbus. Interestingly, however, numerous corneal cross-sections of adult avian tissue exhibited distinct invaginations of epithelium and underlying stroma to form undulating folds at the limbal zone, which perhaps may fulfil a similar function to the palisades.

SEM imaging also revealed a network of collagen with a porous architecture running circumferentially around the corneoscleral junction, which appears similar to the anterior limbal cribriform layer, first identified by Park and colleagues in the human limbus [23]. Using advanced second harmonic generation imaging, Park and associates effectively demonstrated how the “honeycomb” collagen network was occupied by blood vessels circumferentially along the limbal epithelial niche. Our observation of a similar network in the avian cornea adds to the speculation that the presumptive anterior limbal cribriform layer may provide structural support to the limbus with a role in maintaining the surrounding microenvironment occupied by stem cells and vasculature.

SBF-SEM allowed for high-resolution imaging and 3D reconstruction of the adult chicken limbal zone, which identified structures that were anatomically similar to human LCs and PoV as described in the literature [8,9,10,11]. In the human limbus, FSPs are distinct from limbal crypts and are described as finger-like projections of the limbal stroma containing an upward-extending central blood vessel. Shortt and colleagues found that FSPs were surrounded by a compact arrangement of basal cells and, when imaged using SEM, the decellularization of the epithelium surrounding them left remnants of protruding blood vessels visible in the limbal zone [3]. In this study, no such structures were observed using SEM or SBF-SEM; however, serial images of the adult limbus did show blood vessels housed within the upward stromal projections, but along the cross-sectional plane rather than the *en face* orientation.

Within the undulating folds of the adult limbus, especially in the downward projections of epithelium, small circular basal epithelial cells with a high nuclear-to-cytoplasmic ratio, a barely detectable nucleolus, and euchromatin presenting as open DNA were also identified. The location and distinct morphology of these cells, which are indicative of limbal epithelial stem cells, have also been reported in the putative stem cell niche by Dziasko et al. [9] and Yamada et al. [6] in human and rabbit corneas, respectively. In the study by Dziasko and colleagues, small limbal epithelial cells appeared to make direct contact with stromal cells in the human niche. The resolution and contrast of the SBF-SEM images obtained in this study were lower than those in Dziasko’s work, which made it more challenging to discern the nuclear-to-cytoplasmic ratio in basal cells and to see whether these cells were in contact with underlying stromal cell processes. However, cells with a high nuclear-to-cytoplasmic ratio were occasionally seen in the presumptive LCs of the adult chicken cornea. This suggests that these cells may be stem cells, but more work using specific stem cell markers would be required to substantiate this notion. The TEM of the adult limbus was able to substantiate this, with higher-resolution images of a small epithelial basal cell that exhibited a relatively large nucleus compared to its surrounding cytoplasm, in addition to lobed processes of basal cells projecting into the superficial stroma that connect with neighbouring stromal cells.

In the limbal niche of the human cornea, PoV contain a vascular complex; however, unlike with FSPs, the blood vessels within the PoV have a radial orientation (parallel to the PoV) rather than an upward (anterior surface plane) projecting blood vessels. The orientation of blood vessels seen here suggests the vascularized stromal projections seen in the adult avian cornea are more akin to PoVs than FSPs. Interestingly, the anatomical orientation of the presumptive LCs and PoV were seen to run circumferentially along the limbus in the avian cornea as opposed to radially in the human cornea. This apparent difference is unsurprising, as the presentation of the limbal stem niche structures in the human cornea is not constant across all mammalian species. PoV, for example, is reportedly absent in rabbit and rodent corneas but present in porcine corneas [6,24,25]. Also, recent work by our research group has found that in the porcine cornea, multiple circumferential limbal “troughs” are seen in place of the small, discrete LCs that are found in the human limbus [26], emphasizing cross-species differences in the microanatomy of the limbal stem cell niche.

### 4.2. Embryonic Limbal Zone

Late-stage chick embryos were used to investigate the anatomical maturation of niche-related structures. Unlike in the adult limbus, well-defined presumptive LCs and PoV were not observed in either E18 or E16 corneas. Instead, miniature undulating folds were present in their place, which resembled those observed in the rabbit limbus [6]. In a study by Davies et al. [27], fetal corneas from 8.5 to 22 weeks’ gestation were examined to better understand the development of the human limbal niche. This study found that papillae-like structures (PoV) were absent from fetal corneas but were evident in 3-week postnatal specimens. Other limbal stem cell structures also remained unidentifiable in both fetal and neonatal specimens, which suggested that niche structures do not form until after birth [27]. Yeung and colleagues also failed to identify LCs in the cornea of a 4-month-old infant [28], which is supportive of the notion that presumptive limbal niche structures identified in the embryonic avian cornea may not be the same as those seen in mature tissue.

In later embryonic development, the epithelial basement membrane showed distinct differences at the interface between the peripheral cornea and limbus. Basal cells along the peripheral cornea, for example, presented in a uniform and aligned fashion, whereas basal cells at the limbus adopted a highly irregular profile, extending lobed processes into the underlying stroma, thus displaying a similar appearance to that which was reported in the rabbit by Yamada et al. [6]. In the absence of LCs in the rabbit cornea, an irregular profile was viewed to markedly increase the surface area towards the superficial mesenchyme and was suggested by the authors to represent small-scale palisades that broadly fulfilled the same role as those seen in the human limbus [6]. In both the developing and adult avian limbus, it was clear that the basement membrane profile increases the surface area of the interface between the epithelium and underlying stroma; however, as the cornea matures, distinct and pronounced protrusions in the form of folds become more evident to allow for greater interaction with the superficial mesenchyme. The current investigation showed evidence of direct cell–cell interaction between stromal and epithelial cells in the developing avian limbus, a finding also reported by Dziasko et al. [9] and Yamada et al. [6] in relation to the putative limbal stem cell niche in the human and rabbit cornea, respectively. The contacts in the human limbus were seen to form along the “walls” of limbal crypts, whereas the connections observed in the developing chick limbus were seen to form with the lobed processes of basal cells, as is the case in the rabbit limbus.

Incidentally, evidence of matrix structures proximally extending from the epithelial basement membrane was observed in the limbi of late-stage embryonic corneas. These structures, akin to matrix cords, were first described in relation to the dissociation between the connected presumptive lens and corneal epithelium during early ocular development [12,29]. Recent work by our research group has shown these cords to emerge from the central epithelial basal lamina to interface directly with underlying stromal cells and persist throughout development (E6–E18) in the avian cornea [30,31]. Our group speculates that the developing avian cornea may drive mechanotransduction cues through these cell–matrix interactions to detect physical and compositional information about the surrounding corneal environment in order to regulate cell behaviour [30]. In this study, the peripheral cornea was also observed to share similar interactions between emergent cords and stromal cells, previously only seen in the central cornea.

## 5. Conclusions

In recent years, there has been a growing body of research aimed at combating sight-threatening sequelae associated with ocular surface diseases resulting from limbal stem cell deficiency. The corneal limbus is widely accepted as a supportive microenvironment for both epithelial and mesenchymal stem cell populations in the eye. However, the full extent of the milieu’s functional role has yet to be ascertained, particularly with respect to the pathways involved in cell renewal. Here, we describe microanatomical features and cell–cell interactions in the developing and mature avian cornea, which are often comparable with those seen in the human limbal stem cell niche. The documented variability in some aspects of limbal matrix structure is likely to be reflective of naturally occurring inter-species differences [4,5,6,7], but the finding of anatomical structures resembling presumptive PoV and LCs in the avian cornea points to some commonality in the microanatomy of the limbal stem cell niche. The significance of this implication may encourage the use of the chick model in new avenues of research into understanding the formation of a corneal niche and its application in developing new disease models to understand the mechanisms that affect limbal function and dysfunction.

## Data Availability

Data will be made available upon reasonable request.

## Supplementary Materials

The following supporting information can be downloaded at: https://www.mdpi.com/article/10.3390/cells12192334/s1. Video S1: 3D reconstruction of limbal quadrant one in the adult avian cornea from SBF-SEM images. Video S2: 3D reconstruction of limbal quadrant four in the adult avian cornea from SBF-SEM images. Video S3: A 3D reconstruction of stromal blood vessels and red blood corpuscles in the adult avian cornea. Video S4: A 2D flythrough of the limbal zone using SBF-SEM in the E16 cornea. Video S5: A 2D flythrough of the limbal zone using SBF-SEM in the E18 cornea.
